# Small-Pox

**Published:** 1905-07-22

**Authors:** 


### The Hospital.

A Journal of

The Medical Sciences and Hospital Administration.

Vol. XXXVIII.—No. 982. SATURDAY, JULY [illegible], 1905.

### SMALL-POX.

The Conference held at the Mansion House on Tuesday, under the auspices of the Imperial Vaccination League, with a view to the prevention of epidemic small-pox, afforded an opportunity of placing before the public some very remarkable statistics, upon the significance of which it is worth while to dwell. Between February 28th, 1904, and February 25th, 1905, 8,229 cases of small-pox were notified in 315 urban and 94 rural districts in England and Wales, in addition to (possibly single) cases, the number of which was not stated, but which occurred in nine port sanitary districts in the same divisions of the kingdom. In all, cases occurred in 418 districts in England and Wales. In 78 districts the disease was limited to a single case in each; and it is not too much to assume that such a limitation must have been attributable to the manner in which vaccination had been carried out by the local authorities as well as to the general excellence of their administration. Forty-eight districts had two cases each, 23 had three cases each, and 25 had four cases each. From this point the numbers rise somewhat rapidly, the maximum being 560 cases in Dewsbury, followed by 455 in London, 382 in Newcastle-on-Tyne, 373 in Gateshead, 307 in Ravensthorpe, 301 in Oldham, and 262 in Leicester. Numbers between 100 and 200 are furnished by Ashton-under-Lyne, Barnsley, Batley, Bradford (Yorkshire), Derby, Felling, Halifax, Leeds, Manchester, Nottingham, Ossett, Preston, South Shields, Stockport, and Thornhill. The case of Leicester is rendered particularly interesting and instructive by its well-known history. The town has been notorious, not only as one of the chief centres of the agitation against vaccination, but also as a place where the agitators pinned their faith upon general sanitation and isolation as adequate protective influences, and carried these measures to an extreme degree, especially the isolation, which, in the cases of "contacts" is supposed sometimes to have gone considerably beyond legal limits. Notwithstanding this, it was pointed out during the Conference that the cases of small-pox notified for the year amounted to about one in every 800 of the population, a proportion which, if it had been maintained for London, would have given about 5,600 cases instead of the 455 which actually occurred, or to Manchester 674 cases instead of the 131 which occurred there. Leeds, in the same proportion, would have had 531 cases instead of 138, and so on. In instances like that of Dewsbury, where the epidemic bore a very large proportion to the population, it is fair to infer that some very exceptional conditions must have been in operation.

Against the appalling total of 8,229 cases of small-pox in England and Wales in the course of a single year, we have to set the consideration that, as lately shown by the careful inquiry instituted by the Local Government Board, the disease has been absolutely extinguished in the German Empire. It is continually brought over the frontiers, especially from Russia, but it finds no susceptible population upon which to act. The imported cases are placed in ordinary hospitals, with no special precautions as to isolation, and the disease does not and cannot spread. The great towns of Germany are saved the enormous cost of the provisions for isolation which we are compelled to maintain as an insurance against epidemics; and they are not only saved from the deaths, the disablement, and the constitutional weakening of many survivors, which every epidemic occasions among ourselves, but also from the withdrawal of the healthy from useful occupations for the purpose of tending the sick. We submit in this country to an annual tax of thousands of pounds in money, of numerous lives, and of impaired health and diminished productiveness among the survivors, for no conceivable good purpose. The example of Germany is now complete upon every aspect of the question. It has shown, conclusively, that vaccination and revaccination, when rendered realities, afford effective national protection against the disease. It has shown, equally conclusively, that vaccination and revaccination have no injurious influence upon the health of a vast population regularly subjected to them. We have not much cause for gratitude to Germany in the past, and are perhaps likely to have still le[illegible] in the future. It is all the more necessary [illegible] acknowledge that she has turned one of the greatest of English discoveries to her own advantage in a manner which puts us to shame for our neglect of it. The resolution carried at the Mansion House, "that this meeting of Lord Mayors and Mayors,

Lord Provosts and Provosts, and Chairmen of the Urban or Rural District Councils of such towns and localities in Great Britain and Ireland as have suffered collectively from upward of 480 separate epidemics of small-pox within 18 months venture to urge upon His Majesty's Government the great importance of preventing the recurrence of such epidemics by the systematic re-vaccination at school age of all but the children relieved under the Exemption Clause and of those who are for a time excused on the ground of health," is one that is only too likely, in the present state of politics, to be absolutely ineffective; but it will at least remain on record as an expression of enlightened opinion, founded upon very wide and very melancholy experience.

